# The dissemination potential of *Microsporidia MB* in *Anopheles arabiensis* mosquitoes is modulated by temperature

**DOI:** 10.1038/s41598-025-07414-7

**Published:** 2025-08-07

**Authors:** Fidel Gabriel Otieno, Priscille Barreaux, Affognon Steeven Belvinos, Edward Edmond Makhulu, Thomas Ogao Onchuru, Anne Wambui Wairimu, Stancy Mandere Omboye, Cynthia Nyambura King’ori, Bonoukpoè Mawuko Sokame, Anthony Kebira Nyamache, Jeremy Keith Herren

**Affiliations:** 1 International Centre of Insect Physiology and Ecology, Nairobi, Kenya; 2 Department of Microbiology, Biochemistry and Biotechnology, Kenyatta University, Nairobi, Kenya; 3 Department of Physical and Biological Sciences, Bomet University College, Bomet, Kenya; 4 Wits Research Institute for Malaria, University of the Witwatersrand, Witwatersrand, South Africa

**Keywords:** *Microsporidia MB*, Malaria control, Endosymbiosis, Temperature, Dissemination potential, Computational biology, Applied microbiology, Computational science

## Abstract

*Microsporidia MB*, a vertically transmitted endosymbiont of *Anopheles* mosquitoes, shows strong potential as a malaria control agent due to its ability to inhibit *Plasmodium* development within the mosquito host. To support its deployment in malaria transmission reduction strategies, it is critical to understand how environmental factors, particularly temperature, influence its infection dynamics. In this study, we investigated the impact of four temperature regimes (22 °C, 27 °C, 32 °C, and 37 °C) on *Microsporidia MB* prevalence and infection intensity by rearing mosquito larvae under controlled laboratory conditions. Our results demonstrate that elevated temperatures, especially 32 °C, significantly enhance both larval growth and *Microsporidia MB* infection rates. Population growth modeling further indicates that at 32 °C, an infected mosquito population can reach 1000 offspring within 15–35 days, representing a 4.7-, 1.3-, and 1.7-fold increase in dissemination potential compared to 22 °C, 27 °C, and 37 °C, respectively. Although mortality at 32 °C was approximately 20% higher than at 27 °C, this temperature emerged as the most favorable for mass-rearing *Microsporidia MB*-infected larvae. These findings provide the first insights into temperature-mediated dynamics of *Microsporidia MB* and support its potential for scalable implementation in malaria-endemic regions.

## Introduction

Malaria prevention and management are increasingly challenged by global temperature rise^1^ and the growing threat of insecticide resistance, particularly in low- and middle-income countries in sub-Saharan Africa^2,3^. Climate change has been shown to accelerate insecticide resistance, further undermining the effectiveness of current insecticide-based control strategies^4–7^. As a result, the global malaria burden continues to increase, with 249 million cases in 2022, up from 231 million cases in 2015^8^. The World Health Organization’s goal of reducing malaria cases and mortality by 90% by 2030 cannot be achieved through insecticide-based methods alone^9^. While insecticide-treated nets and indoor residual spraying of insecticides remain central to vector control, novel and complementary malaria control innovations are urgently required^10^. These new tools must be assessed in the context of their interactions with environmental conditions, vector ecology and existing interventions^8^.

*Microsporidia MB*, a naturally occurring symbiont, shows strong promise as a complementary malaria control strategy due to its ability to inhibit *Plasmodium* parasite development in *Anopheles* mosquitoes^11,12^. It has been identified in multiple *Anopheles* species including *An. arabiensis, An. gambiae, An. coluzii*, and across diverse geographical regions^12–14^, *Microsporidia MB* spreads via vertical and horizontal transmission routes^11^, and its prevalence displays seasonal variation, with higher infection rates typically observed following peak rainfall^12^, consistent with patterns seen in other microsporidians in *Anopheles* mosquitoes^15^. However, the influence of temperature and climate on its infection dynamics remains poorly understood and is crucial for maximizing its effectiveness as a vector control tool.

Temperature plays a pivotal role in shaping microsporidian biology, with high temperatures and low humidity generally promoting increased infection intensity and spore production^15–21^. Environmental conditions also strongly influence mosquito physiology, survival, reproduction, and development^15,22–30^. In other insect species, such as the stinkbug pest *Nezara viridula*, higher temperatures have been shown to disrupt the gut microbiome and reduce host fitness^31^. For *Microsporidia MB,* transmission is intensity-dependent^11,12^ and infection levels are modulated by factors such as mosquito age and blood feeding history^12,32^. Thus, temperature may exert a profound effect symbiont proliferation and dissemination, presenting both opportunities and limitations for its use in malaria control^16^.

In this study, we investigated the influence of temperature on the dissemination potential of *Microsporidia MB* in *An. arabiensis* mosquitoes collected from Ahero irrigation scheme, where genome sequencing identified a single *Microsporidia MB* isolate^33^. By evaluating larval development time, survival rates, and infection intensity under controlled temperatures regimes (22 °C, 27 °C, 32 °C, and 37 °C), we developed a population growth model to simulate the spread of *Microsporidia MB*-infected *An. arabiensis* mosquitoes. The reference temperature for rearing this species is 27 °C^34^, with 22 °C and temperature above 35 °C mark critical thresholds for development^35^. Our model estimates how rapidly *Microsporidia MB* can establish within a mosquito population, offering insights into the optimal conditions for maximizing its amplification. By predicting the symbiont’s dissemination dynamics, this work supports efforts to evaluate and optimize *Microsporidia MB* for scalable implementation in malaria-endemic regions.

## Results

### Quantification of *Microsporidia MB*

The vertical transmission rate of *Microsporidia MB* in the *Anopheles arabiensis* iso-female lines used for this study was 43%, indicating that approximately 43% of offspring from *Microsporidia MB*-infected (MB+) females inherited the symbiont. The remaining 57% were uninfected, either as offspring of MB + female or uninfected (MB−) females.

#### Pupation rate

**Fig. 1:**
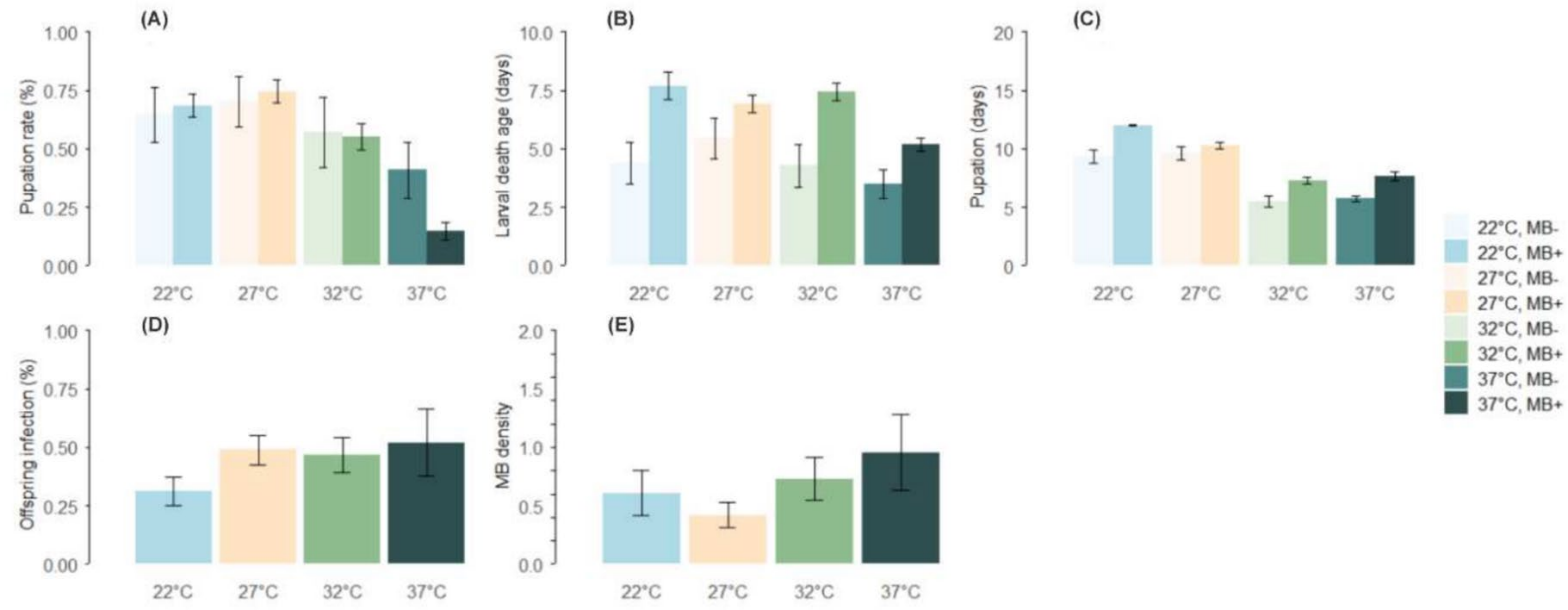
Panel representing the average (**A**) Pupation rate: The pupation rates of the offspring from MB+ female *An. arabiensis* differed significantly by temperature, with all other temperatures significantly different from 27 °C. The highest pupation rate was observed at 27 °C, 74.4 (70.14–79.56) %; then 22 °C, 68.4 (63.37–73.37) %; then 32 °C, 55.8 (49.84–60.95) % and low at 37 °C, 14.6 (10.77–18.40) % (Tukey for MB + female *An. arabiensis*: p_22 °C–27 °C_ = 0.001; p_27 °C–32 °C_ = 0.137, p_27 °C–37°C_ < 0.001; p_32 °C–37°C_ < 0.001; p_22 °C–32 °C_ > 0.05; *χ*^2^ = 29.39 df = 3, *p* < 0.001). (**B**) Age at death: MB− female *An. arabiensis*’ offspring died two days earlier on average than the offspring from the MB+ female *An. arabiensis* regardless of the temperature [MB− female *An. arabiensis*: 4.2 (3.62–4.86) days; MB+ female *An. arabiensis*: 6.4 (6.07–6.66) days] (*χ*^2^ = 18.66, df = 1, *p* < 0.001), except at 27 °C where we found no significant differences between the female *An. arabiensis*’ groups (Tukey 27 °C: p_MB+=/MB−_ > 0.05); *χ*^2^ = 9.98, df = 3, *p* = 0.02). **(C)** Time to pupation: In offspring coming from MB- female *An. arabiensis*, the time to pupation did not vary between 22 and 27 °C (Tukey: p_22 °C–27 °C_ > 0.05), but we observed a two days difference in offspring coming from MB+ female *An. arabiensis* reared in these temperatures [22 °C: 12.0 (11.70–12.31) days; 27 °C: 10.2 (9.93–10.48) days; 32 °C: 7.4 (6.89–7.42) days; 37 °C: 7.6 (7.25–8.04) days, (Tukey: p_22 °C–27 °C_ < 0.001; *χ*^2^ = 21.89, df = 3, *p* < 0.001). (**D**) Infection rate: The highest infection rate was recorded at 37 °C (52.1%) followed by 27 °C (48.7%), 32 °C (46.7%) and 22 °C (30.8%), there was no significant difference in pupation across all temperature regimes (Tukey: estimate_27 °C–22°C_ = 0.68, p_27 °C–22 °C_ = 0.001; p_27 °C–32°C_ and p_27 °C–37 °C_ > 0.05; *χ*^2^ = 9.99, df = 1, *p* = 0.001) (**E**) M*icrosporidia MB* intensity: The lowest *Microsporidia MB* intensity in the offspring was observed at 27 °C which was significantly different from the intensity in those maintained at 37 °C [22 °C: 2.2 (0.34–4.01) ratio_MB18S/S7_; 27 °C: 1.0 (0.55–1.43) ratio_MB18S/S7_; 32 °C: 2.2 (1.27–3.11) ratio_MB18S/S7_; 37 °C: 2.55 (1.28–3.75) ratio_MB18S/S7_; (Tukey: p_22 °C–37 °C_ < 0.001; p_27 °C–37 °C_ = 0.001; *χ*^2^ = 19.40, df = 3, *p* < 0.001)]. At 27 °C and 37 °C, the development time was negatively correlated to *Microsporidia MB* intensity in the offspring. At 27 °C faster pupation led to a 45% increase in *Microsporidia MB* intensity, while at 22 °C, delayed pupation increased intensity by the same amount [y_(27)_ = 9.7 − 0.467x, r^2^ = 0.12; y_(22)_ = 11.4 + 0.311x, r^2^ = 0.13; y_(37)_ = 7.75 − 0.252x, r^2^ = 0.23]. The intensity at 32 °C was unaffected by pupation time [y_(32)_ = 7.33 + 0.00623x, r^2^ < 0.01]. (data log transformed for better data visualization) in offspring coming from non-infected (MB-, lighter colours) and infected female *An. arabiensis* (MB+ , darker colours) reared in 4 temperature treatments: 22 °C (blue bars), 27 °C (tan bars), 32 °C (green bars) or 37 °C (emerald bars). Average pupation rates are calculated out of the total count of offspring, average larval death ages are calculated for larvae that died, average times to pupation were calculated for all individuals that pupated, offspring infection rates were calculated for all individuals coming from infected female *An. arabiensis*, and average *Microsporidia MB* intensities were calculated for all MB infected offspring. The error bars show 95% confidence intervals.

Across all temperature treatments, the mean pupation rate was 54.1 (95% confidence interval: 51.60–56.59) %. At 27 °C, the pupation was significantly higher (73.8% 69.44–78.14) compared to 32 °C (56.1%, 50.92–61.13) and 37 °C (19.0%, 15.12–22.86), but not significantly different from 22 °C (67.8%, 63.16–72.36). Tukey post-hoc tests confirmed significant differences between 27 °C and both 32 °C and 37 °C (p_27 °C–32 °C_ and p_27  °C–37  °C_ < 0.001), but not between 27 °C and 22 °C (p_27 °C–22 °C_ > 0.05) [Model statistics: *χ*^2^ = 153.95, df = 3, *p* < 0.001; Fig. 1A].

Offspring of MB− females had higher overall pupation success (58.5%, 52.29–64.73) than offspring of MB + female (53.3%, 50.56–56.00), with the strongest effect observed at 37 °C. At this temperature, offspring from MB− females exhibited threefold higher pupation success than those from MB+ females [MB−: 40.9% (0.29–0.53), MB+: 14.6% (10.77–18.40); Tukey post-hoc at 37 °C: p_MB-/MB+_  < 0.001; all other comparisons: *p* > 0.05; model statistics: *χ*^2^ = 11.26, df = 1, *p* < 0.003].

#### Larval mortality age

Larvae that failed to pupate died on average 6.1 days post hatching (95% CI: 5.79–6.33). Larvae reared at 37 °C died significantly earlier than at other temperatures [22 °C: 7.1 days (6.17–8.02); 27 °C: 6.6 days (5.93–7.35); 32 °C: 7.1 days (6.53–7.60); 37 °C: 5.0 days (4.69–5.27)]. Tukey post-hoc tests showed significant differences between 37 °C and all other treatments [all Tukey hoc tests *p* < 0.001; model statistics: *χ*^2^ = 39.79, df = 3, *p* < 0.001; Fig. 1B].

#### Time to pupation

Larvae took on average 9.6 days (95% CI 9.41–9.80) to pupate. Offspring of MB- female pupated significantly faster than those of MB+ females [MB−: 8.1 days (7.67–8.47); MB+: 9.9 days (9.71–10.13); model statistics: *χ*^2^ = 56.87, df = 1, *p* < 0.001]. Among MB+ females, MB+ offspring developed faster than their MB− siblings [MB+: 9.1 days (8.85–9.40); MB− from MB+: 10.2 days (9.93–10.52); *χ*^2^ = 50.92; model statistics: df = 2, *p* < 0.001; Supplementary Fig. 1]. This effect was most pronounced at 27 °C [MB+ offspring: 9.4 days (9.06–9.83); MB− from MB+: 11.1 days (10.66–11.46); Tukey post-hoc test: *p* = 0.02; model statistics: *χ*^2^ = 21.92, df = 6, *p* = 0.001]. At 22 °C, 32 °C, and 37 °C, MB+ and MB−_ offspring from MB+ females showed no significant differences in development time (all Tukey tests *p* > 0.05).

Overall, development time decreased with increasing temperature. Larvae reared at 32 °C and 37 °C [7.1 days (6.89–7.42) and 6.96 days (6.62–7.30), respectfully] pupated 36% faster than those at 27 °C (10.1 days, 9.86–10.36) and nearly 50% faster than at 22 °C (11.6 days, 11.29–11.88). All pairwise comparisons were significant, except between 32 and 37 °C [for all *p* < 0.001, except p_32 °C–37 °C_ > 0.05; model statistics: *χ*^2^ = 137.95, df = 3, *p* < 0.001; Fig. 1C].

#### Infection rate in offspring

Infection rates varied significantly with temperature. Infection was lowest at 22 °C (30.8%, 24.45–37.22) and highest at 37 °C (52.1%, 37.95–66.21). At 27 °C the infection rate was 48.7% (42.43–55.07), significantly higher than at 22 °C but not significantly different from 32 °C (46.7%, 39.06–54.28) [Tukey post hoc tests: p_27 °C–22 °C_ = 0.68, p_27 °C–22 °C_ = 0.001; p_27 °C–32 °C_ and p_27 °C–37 °C_ > 0.05; model statistics: *χ*^2^ = 9.99, df = 1, *p* = 0.001; Fig. 1D]. Offspring infection levels were positively correlated with maternal *Microsporidia MB* intensity (Supplementary Fig. 2).

#### *Microsporidia MB* infection intensity in offspring

Among MB + offspring, the relative *Microsporidia MB* intensity (normalized to the *Anopheles* S7 gene) was 1.7 (95% CI 1.19–2.23). Higher infection intensity was associated with faster development (*χ*^2^ = 6.56, df = 1, *p* = 0.01). This relation however varied by temperature [Tukey post hoc tests: p_22 °C–27 °C_ < 0.001; p_27°C-37 °C_ = 0.06; p_27 °C–32 °C_ = 0.001; model statistics: *χ*^2^ = 17.45, df = 3, *p* < 0.001; Fig. 1E)]. Additionally, female *An. arabiensis* with a transmission rate > 50% produced offspring with greater *Microsporidia MB* intensity (Supplementary Fig. 3).

### Modelling the *Microsporidia MB* dissemination potential

**Fig. 2:**
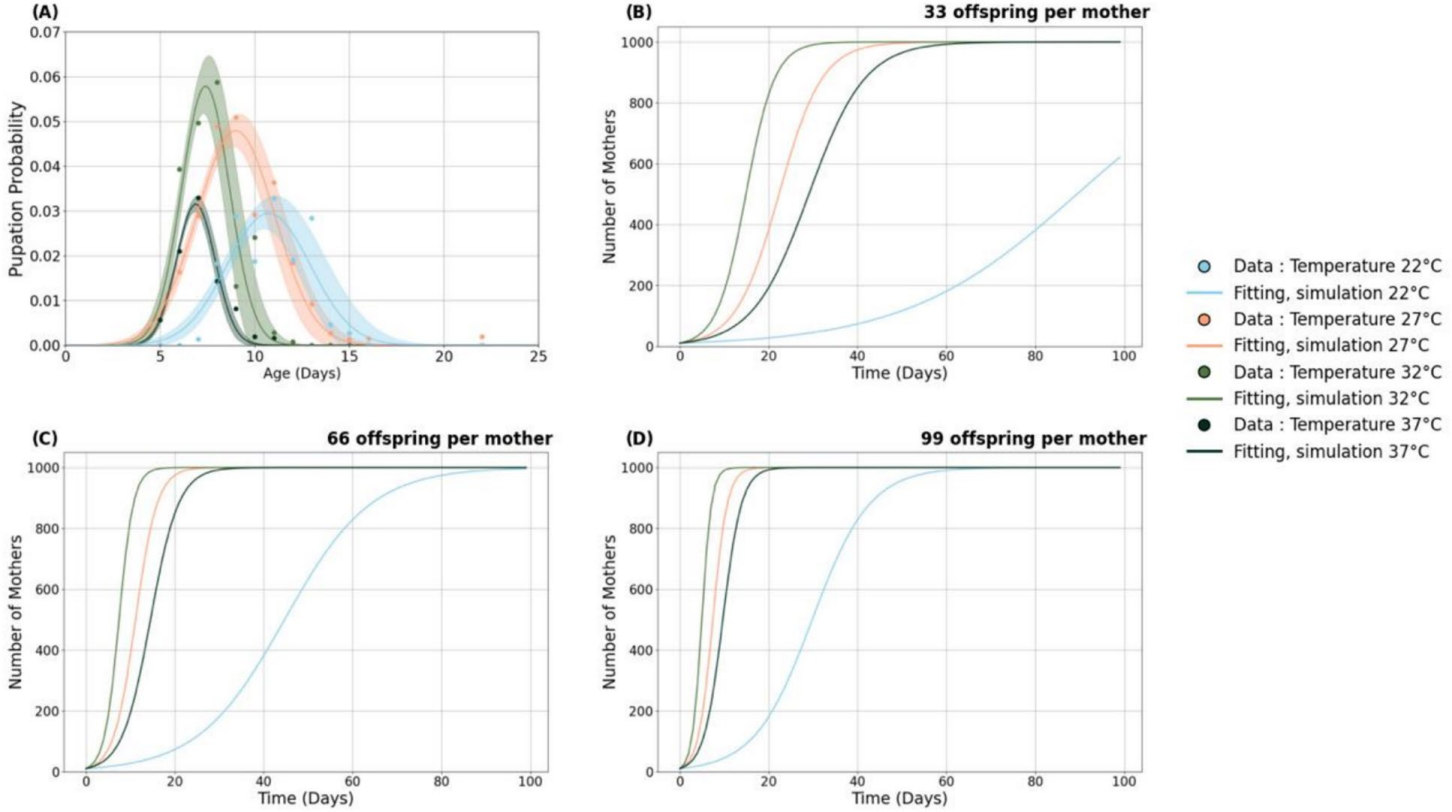
Panel representing the effects of 4 different temperature treatments: 22 °C (blue colour), 27 °C (tan colour), 32 °C (green colour) or 37 °C (emerald colour) on (**A**) The probabilities ${\mathbb{P}}\left( {T,x} \right)$ modelled using a gaussian function that an offspring is infected, survives to age *x*, and pupates at age x and (**B**–**D**) represent the population growth of MB+ offspring starting with an initial population of 10 MB+ female *An. arabiensis* and progressing over a 100-day period across the 4 four temperature treatments considering different fecundity levels; 33, 66 and 99 offspring per female *An. arabiensis*, respectively. Each growth curve incorporates a sex ratio of 0.5 (indicating an equal proportion of female offspring), a generation cycle and is capped by a carrying capacity *K* = 1000, illustrating how temperature affects the speed and likelihood of reaching the target population over time.

Using experimental data, we developed a mathematical model to predict the spread of *Microsporidia MB* in *Anopheles arabiensis* populations under different temperature regimes. The time required to reach a target population of a thousand MB+ offspring from an initial population of ten female *An. arabiensis* was significantly influenced by temperature. As temperature increased, the mean age at pupation decreased, and the probability of successful pupation increased (Fig. 2A).

The probability that an offspring is infected, survives to age *x*, and pupates at age *x* given temperature *T* was modelled using fitted parameters: *A* (scaling factor), *mu (µ)* (mean age of pupation), and *sigma (σ*) (spread of the pupation age). This probability incorporated the temperature-dependent likelihood of infection, the chance of survival to age *x* given infection status, and the probability of pupating at that specific age. The model’s accuracy was validated by high *R*^2^ values, especially at higher temperatures, indicating a strong fit to the observed data. At 22 °C, the normalization constant *A* was 0.02942, the mean pupation age *mu* was 10.73 days, and the standard deviation *sigma* of 2.20. This produced a mean squared error *(MSE)* of 0.00003 and an R^2^ value of 0.794, indicating a moderate fit. At 27 °C, *A* increased to 0.04785, *mu* decreased to 8.99, and *sigma* was 2.06, with an MSE of 0.00003 and a stronger fit at R^2^ = 0.917. At 32 °C, A was 0.05782, *mu* dropped to 7.40, and sigma narrowed to 1.27, yielding an MSE of 0.00005 and an R^2^ of 0.874. Finally, at 37 °C, *A* was 0.03147, *mu* was 6.88, *sigm*a was 1.02, with the best model fit (*MSE* of 0.00000, R^2^ of 0.970).

Population growth simulations across different temperatures and fecundity levels revealed that both parameters strongly increased growth rates and the time needed to reach a population of 1000 *MB*+ offspring. The optimum temperatures 27 °C and 32 °C consistently led to rapid population growth, with 1000 *MB*+ offspring reached within 15–48 days across all fecundity levels. Specifically, at a fecundity level of 33 offspring per female *An. arabiensis* (Fig. 2B), populations reached 1000 *MB*+ offspring within approximately 48 days at 27 °C, 35 days at 32 °C, and 62 days at 37 °C. At 22 °C, the population did not reach 1000 *MB*+ offspring within the 100-day period.

Increasing fecundity to 66 offspring per female *An. arabiensis* (Fig. 2C) led to faster population growth, with 1000 *MB*+ offspring reached by day 25 (27 °C), day 20 (32 °C), and day 35 (37 °C). At 22 °C, the average time to reach 1000 MB+ offspring remained 100 days. At the highest fecundity of 99 offspring per female (Fig. 2D), the population reached 1000 MB+ offspring by day 17 at 27 °C, day 15 at 32 °C, and day 25 at 37 °C. At 22 °C, the population reached the target number by approximately day 65.

To further investigate the influence of fecundity variability, we conducted Monte Carlo simulations with 1000 iterations, incorporating a 10% variation in offspring number (Supplementary information, Fig. 4). These simulations confirmed that temperature, rather than variability in offspring number, was the primary factor influencing the establishment and growth of MB + populations. Across all levels of offspring variability, 32 °C consistently emerged as the optimal temperature for rapid MB+ population expansion.

## Discussion

*Microsporidia MB* is a symbiont of *Anopheles arabiensis* that is transmitted both vertically (from female to offspring) and horizontally (via sexual transmission)^11,12^. Vertical transmission occurs through infection of developing eggs via stem cell division, allowing the symbiont to remain intracellular. In contrast, horizontal transmission is thought to require spore formation and the exit of the symbiont from host cells. While vertical transmission is typically highly efficient, some offspring appear to lose the infection during juvenile developments^36^.

This study investigated how temperature influences the prevalence and spread of *Microsporidia MB* during the aquatic stages of *An. arabiensis* development. In this study, we investigated the impact of temperature on *Microsporidia MB* prevalence and dissemination potential during aquatic stages of *Anopheles arabiensis* development. Our results demonstrate that 32 °C is the optimal temperature for maintaining high symbiont intensity and supporting rapid population growth of MB+ mosquitoes. This contrasts with the standard rearing temperature of 27 °C commonly used for *Anopheles arabiensis* (69). While both MB+ and MB− mosquitoes survived better at 27 °C, the MB + mosquitoes grew more rapidly at 32 °C due to accelerated larval development time and elevated infection intensity. Therefore, rearing temperatures between 27 to 32 °C, particularly closer to 32 °C, appear more favourable for mass production of MB+ mosquitoes for symbiont-based malaria control. However, the malaria-blocking potential of MB+ reared at different temperatures remains to be evaluated.

The offspring used in this study were derived from female wild-caught *Anopheles arabiensis* mosquitoes, which typically exhibit lower fecundity (33–66 offspring per female) when brought to laboratory settings^37–39^. This highlights the importance of considering ecological variability and natural mosquito populations when designing vector control interventions^40^. Temperature emerged as a critical factor influencing both MB+ prevalence and mosquito population dynamics. The strong correlation between temperature and MB+ population growth, supported by high R^2^ values (0.79396–0.97048), suggests that our logistic growth model effectively captures the dynamics of MB+ mosquito proliferation across thermal environments.

As poikilotherms, mosquitoes are highly sensitive to temperature, which affects traits such as longevity, fecundity, biting rate and development of immature stages of mosquitoes, are strongly influenced by temperature^41^. Consistent with previous studies^42,43^, we found that higher temperatures accelerated larval development but pupation rates, regardless of infection status. Notably, larvae from MB+ females generally developed more slowly than those from MB− females. However, when separating infected and uninfected from MB + mothers, we observed that MB+ offspring observed that MB+ offspring actually developed on day faster than their MB- siblings at 27 °C. This findings aligns with earlier laboratory and semi-field studies where temperature was not tightly controlled^12,13,44^.

The distribution and prevalence of certain microsporidian genera, such as *Enterocytospora, Microsporidium* and *Vairimorpha*, are positively associated with increasing environmental temperature^15^. Similarly, in our study, both *Microsporidia MB* prevalence and infection intensity rose with temperature. Although the mechanisms behind this trend remain unclear, temperature-dependent differences in maternal transmission efficiency, symbiont replication, or host immune response could all contribute. Thus, temperature strongly influences the symbiont’s transmission potential and persistence.

We acknowledge that our study does not resolve the mechanism behind differential MB+ prevalence across temperatures. Potential explanations include temperature-driven loss of infection during larval development, differential mortality associated with infection, or unmeasured forms of horizontal transmission. This limitation underscores the need for further targeted studies. Similar phenomena have been observed in *Drosophila*, where higher temperatures promote the proliferation of *Wolbachia* (e.g., *wMelOctoless* and *wMelPop),* increasing their pathogenic effects^43,45^, leading to greater fitness costs associated with pathogenic variants^46^. Conversely, lower temperatures reduce Wolbachia titres, diminishing vertical transmission, as observed with *wYak* in *D. Yakuba*.

In our study, 22 °C produced the lowest MB+ prevalence, suggesting that symbiont loss following vertical transmission is more likely at cooler temperatures^36^. Lower temperatures were also associated with slower development and higher late-stage larval mortality. While speculative, this pattern raises the possibility that *Microsporidia MB* might favour spore formation of larva-to-larva transmission under cold stress. However, no direct evidence of larva-to-larva transmission has been observed to date. Still transmission plasticity is known in other microsporidia^47–49^, with some species switching between vertical and horizontal routes depending on environmental stress^50–52^.

The symbiotic relationship between *Anopheles arabiensis* and *Microsporidia MB* is likely shaped by trade-offs between symbiont persistence, host development, and environmental conditions. Although we did not directly assess how temperature affects the balance between vertical and horizontal transmission, we hypothesize that low temperatures, which prolong larval development and increase physiological stress, could favor spore formation^53,54^. In related systems, such as *Edhazardia aedis*, horizontal transmission becomes more prominent under resource-limited conditions that extends development time^55,56^.

Interestingly, the highest MB+ infection intensity and prevalence were observed at 37 °C. This suggests that elevated temperatures may accelerate symbiont replication, potentially reducing host capacity to clear infections. Although 37 °C is the upper thermal limit for adult *Anopheles gambiae* development^57–59^, *An. arabiensis* larvae may tolerate higher temperatures. The reduced pupation rate observed in offspring from MB+ female at 37 °C, compared to those from MB- females, may indicate a thermal beyond which the symbiont replication becomes detrimental to the host. Excessive heat could damage the symbiont or the host, increasing mortality in MB+ individuals^15^. Similar thermal mismatch effects have been observed in other host-symbiont systems, such as *Daphnia magna* infected with *Pasteuria ramose,* where heat stress impairs host resistance^60^. This aligns with the thermal mismatch hypothesis, which posits that cooler-adapted hosts are more vulnerable to infections from warmer-adapted parasites when exposed to warmer temperature^61^.

We also hypothesize that higher larval mortality at higher temperatures could enhance spore formation and facilitate horizontal transmission. This may explain both the elevated MB+ prevalence and the heightened mortality under hot rearing conditions. Analogous patterns have been observed in *Wolbachia*-infected *Aedes aegypti,* where exposure to 37 °C instead of from 26 °C reversed the infection and blocks vertical transmission^61^.

Larval rearing conditions are known to influence adult mosquito traits such as longevity, fecundity, and overall vector competence^28,62^. Our study focused on juvenile stages, limiting our ability to assess downstream effects on adult fitness or malaria transmission. Nonetheless, it is well established that high temperatures can reduce adult lifespan and fecundity^57^, as well as decrease body size and egg hatch rate^9,27,63^. The relationship between body size and longevity is complex, depending on larval nutrition and temperature^28^, both of which also modulate malaria transmission potential^9,64^. We hypothesize that a similarly complex relationship exists between temperature, *Microsporidia MB* infection, and mosquito survival, warranting further investigation.

While *Microsporidia MB* infection intensity is influenced by nutrition^13,32^, we assumed equal food availability across temperature treatments. However, larval density and food availability are known to interact with temperature to affect mosquito development and fecundity^55,56^. Future study should examine how nutritional conditions impact MB+ mosquito performance, especially given our findings that the number of offspring produced affects the establishment of MB+ populations. At 22 °C, populations did not expand with 33 offspring per female, but populations reached high prevalence with 99 offspring per female even under suboptimal temperatures (22 °C and 37 °C). This highlights the importance of considering both temperature and fecundity in models of *Microsporidia MB* dissemination.

## Conclusion

Although 27 °C is the standard laboratory rearing temperature for several *Anopheles species* including *An. arabiensis* and *An. gambiae s.s.*^37,65,66^, it is not optimal for the spread of *Microsporidia MB*. Our results show that 32 °C supports higher infection intensity, faster larval development, and more rapid population growth, despite reduced mosquito survival at 27 °C. These findings support previous observations that warmer temperatures enhance MB+ mosquito propagation, even with increased mortality risks.

By identifying temperature conditions that favor MB+ establishment and proliferation, this study contributes to the development of effective *Microsporidia*-based malaria control strategies. It also underscores the importance the importance of incorporating environmental variables, especially temperature, into assessments of microbial control methods and into ecological studies of symbiont distribution and transmission. Understanding how climate conditions shape *Microsporidia MB* spread will be critical for predicting the success of MB+ mosquito releases across diverse geographical regions.

## Material and methods

### Experimental design

#### Mosquito collection

1583 larvae used in this study were obtained from 17 field caught gravid *Anopheles arabiensis* female collected via mouth aspiration from Kigoche village (00° 34′ S, 34° 65′ E) in the Ahero irrigation scheme, Kenya and transported to the International Centre of Insect Physiology and Ecology (ICIPE)-Duduville campus in Nairobi, Kenya. During collection, a torch was used to locate *Anopheles gambiae s.l.* indoors on the walls of muddy houses. This was guided by identification protocol illustrated in^67^; the morphological traits used were resting position, characteristics of wings and abdomen. Abdomens of the collected gravid females were morphologically examined and those observed as engorged, and dark were considered gravid. MB+ females used in these experiments were selected over three different collection timepoints. In September 2022, 1123, gravid field collected female *An. arabiensis* were screened for presence of *Microsporidia MB*, 180 were positive resulting in a 16.03% prevalence in the field (5 MB+ females were used for this experiment , offspring n = 404). In November 2022, 399 mosquitoes were screened, 142 were positive for *Microsporidia MB*, this recorded a prevalence of 35.58% of the symbiont in the field (5 MB+ females, offspring n = 511). In July 2023, 565 mosquitoes were screened, 75 were positive for the symbiont resulting to 13.27% prevalence in the field (7 MB+ females, offspring n = 624). The gravid females were placed in 1.5 ml micro-centrifuge tubes containing 1 cm by 1 cm Whatman filter paper to allow egg laying following the methods described in^11–13,32,43^. After oviposition, they were screened for species ID^64^ and the presence of *Microsporidia MB*^11,12,32^ using PCR.

#### Larval rearing

Eggs from *Microsporidia MB* positive and negative female *An. arabiensis* were separated into larval trays with around 300 ml of deionised water to hatch. In three replicates, stage one (L1) larvae from the same MB+ female *An. arabiensis* were randomly and in equal number split into four temperature treatments: A total of 444, 515 and 624 L1 larvae were used to set up replicates one, two and three of the experiments. L1 larvae from each MB+ female *An. arabiensis* were divided into four equal proportions and put in four different larval trays. L1 larvae from MB− female *An. arabiensis* was also put in a separate larval tray for each of the temperature regimes. We, therefore, had four larval trays per each MB+ and MB− female *An. arabiensis*, each tray per temperature regime. One MB− female *An. arabiensis* was used per replicate. This was due to limited space in the incubators. We set temperature 22 °C using insect growth chamber since it supported low temperature settings. Trays for temperature 27 °C were put in an isolated room with control ambient room temperature of 27 °C. We used small incubators to set experiments for temperatures 32 °C and 37 °C, this is because these incubators could only support temperature settings above 30 °C. The number of larvae per tray in the for the MB+ female *An. arabiensis* were dependent on the amount of offsprings produced by each female *An. arabiensis* (the data of larvae per tray in each temperature regime has been attached for reference). In MB− female *An. arabiensis*, 23, 25 and 18 L1 larvae were used per each tray in each temperature regime for replicates one, two and three respectively. The larvae were fed on a pinch of Tetramin baby fish food throughout their development until pupation. We monitored daily larval mortality, rate and date of pupation of each pupa.

#### Quantification of *Microsporidia MB*

The ammonium acetate protein precipitation method was used for DNA extraction from offsprings of MB+ female *An. arabiensis*^68,69^. Whole pupae were homogenised in 50 µl of phosphate buffered saline (PBS), incubated at 56 °C for 1 h in 300 µl of cell lysis buffer then we precipitated out proteins using 100 µl protein precipitate while incubating the samples in ice for 30 min. The supernatant was centrifuged for 20 min at 14,000 revolutions per minute then transferred to 300 µl of isopropanol, the samples were inverted 100 times to allow the reagents mix before centrifuging at 14,000 revolutions per minute for 1 h to remove excess salt. To obtain a clean DNA, we poured out the resulting supernatant then added 300 µl of ice cold 70% ethanol, inverted the samples 50 times then centrifuged at maximum speed of 14,000 revolutions per minute for 30 min to remove excess salts. The resultant DNA was air dried under the biosafety cabinet overnight before elution in 60 µl of nuclease free water^11,12,32,43^.

All pupae collected from the experimental group (offspring of MB+ female *An. arabiensis*) were screened to identify those infected with *Microsporidia MB* using conventional PCR^11,12,32^. We measured the *Microsporidia MB* infection rate in the collected G_0_ female *An. arabiensis* and offspring as well and quantified *Microsporidia MB* density through relative quantification using qPCR. Partial *Microsporidia MB* 18 s gene region from each DNA sample was amplified using specific 18 s primers (MB18SF: CGCCGG CCGTGAAAAATTTA and MB18SR: CCTTGGACGTG GGAGCTATC)^11–13,32,43^. The gene was then amplified in an 11 µl reaction volume of a mixture containing 0.5 µl of 5 pmol/µl reverse and forward primers, 2 µl HOTFirepol Blend Master Mix Ready-To-Load (Solis Biodyne, Estonia), 6 µl of nuclease-free PCR water and 2 µl of DNA template. The amplification was achieved under the following conditions: initial denaturation at 95 °C for 15 min, denaturation at 95 °C for 1 min for 35 cycles, annealing at 62 °C for 30 s, a further extension for 30 s at 72 °C, and finally, final elongation for 5 min at 72 °C. To quantify the level of infection, samples positive for *Microsporidia MB* were subjected to relative qPCR analysis using MB18SF/MB18SR primers normalised with the reference host-keeping gene for the *Anopheles* ribosomal s7 gene (S7F: TCCTGGAGCTGGAGATGAAC and S7R: GACGGGTCTGTACCTTCTGG). Since the ribosomal protein S7 is a highly conserved gene in *Anopheles* mosquitoes, its expression levels are stable across different conditions and tissues, making it a reliable internal control for qPCR experiments^70^. The qPCR reaction mixture consisted of 11 µl reaction volume containing 0.5 µl of 5 pmol/µl reverse and forward primers, 2 µl HOT FIREPol^®^ EvaGreen® 416 HRM no ROX Mix Solis qPCR Master mix (Solis Biodyne, Estonia), 6 µl of nuclease-free PCR water and 2 µl of DNA template. The amplification was achieved under the following conditions: initial denaturation at 95 °C for 15 min, denaturation at 95 °C for 1 min for 35 cycles, annealing at 62 °C for 60 s, and a further extension for 45 s at 72 °C. The PCR was carried out in a proflex cycler, and the qPCR was carried out in a MIC qPCR cycler (BioMolecular Systems, Australia). The MB18SF/MB18SR primers were used to confirm samples with the characteristic *Microsporidia MB* melt curve^11–13,32,43^.

### Statistical analysis

We analysed the pupation rate and age at death using Mixed-Effects Cox Models and the R “coxme” package^71^. The mean development time for the pupated larvae was analysed using the linear mixed-effects model using the “lme4” package. We analysed the infection rate and *Microsporidia MB* intensity using binomial and gaussian logistic mixed-effect model (GLMMs) and glmmTMB package. In all models, the temperature treatments, the G_0_ female *An. arabiensis*' infection status, and their interactions were included as fixed terms, and the time of capture in the field was included as a random effect. In addition, the development time model also looked at the interaction between temperature treatments and infection status in offspring (*Microsporidia MB* negative offspring coming from un-infected colonized female *An. arabiensis*, *Microsporidia MB* positive offspring coming from field-collected infected G_0_ female *An. arabiensis* and *Microsporidia MB* negative coming from field collected infected G_0_ female *An. arabiensis*). Individuals that pupated were excluded from the age-at-death analysis. Individuals who died were excluded from the development time and infection status analysis. The *Microsporidia MB* intensity analysis (log transformed for better data visualisation) excluded uninfected pupae, and we used temperature treatments and transmission groups (0–33%, 33–66%, or 66–99% transmission from mother to offspring) as interaction terms in the model. We used the Tukey post-hoc test and “means” function to perform multiple comparisons among the infection status and temperature treatments^72^. *P values* for comparisons among treatments have been stated before the overall *p values* for each model done. Statistical analysis was performed using R statistical software version 4.1.2 and R Studio^73^.

### Modelling the *Microsporidia MB* dissemination potential

After obtaining experimental data on infection rates, development, and survival, we used these parameters to develop a mathematical model predicting *Microsporidia MB* dissemination in *Anopheles arabiensis* populations under different temperature conditions. To express the probability that an L1 offspring coming from MB+ female *An. arabiensis* is infected, survives to age *x*, and pupates at age *x* given temperature *T*, we combined the conditional probabilities:

*P*(infected $\cap$ survives to age × $\cap$ pupates at *x| T*) = P(infected| *T*). *P* (survives to age *x* |infected*, T*)*. P* (pupates at *x*| infected, *T*).

$$P(infected| T) = \frac{{{\text{Number of infected larvae at temperature }}T{ }}}{{{\text{Total number of larvae at temperature }}T}}$$

$$P(survives to age x |infected, T) = \frac{{{\text{Number of infected larvae that survive to age }} \times {\text{ at temperature }}T{ }}}{{{\text{Total number of larvae at temperature }}T}}$$

$$P(pupates at x| infected, T) = \frac{{{\text{Number of infected larvae that pupate to age }} \times {\text{ at temperature }}T{ }}}{{{\text{Total number of larvae that survive to age }} \times {\text{ at temperature }}T}}$$

Using the Gaussian function, the probability is given by:

$${\mathbb{P}}\left( {{\text{T}},{\text{x}}} \right) = P(infected \cap survives to age x \cap pupates at x| T) = Ae^{{ - \frac{{\left( {x - \mu } \right)^{2} }}{{2\sigma^{2} }}}} ,$$

$$0 < x < \infty$$.

This formula considers the conditional dependencies based on infection status and temperature, providing a logical path to estimate the combined probability.

A continuous logistic model was chosen to provide a smooth and accurate representation of mosquito population growth, reflecting natural, gradual changes without the constraints of fixed time intervals required by discrete models. This continuous approach allows precise population estimates at any point in time, making it ideal for understanding temporal growth rates and incorporating stochastic variability to reflect environmental influences on fecundity. The logistic growth equation:

$$\frac{{{\text{dN}}\left( {\text{t}} \right)}}{{{\text{dt}}}} = {\text{F}}.{\text{r}}.{\mathbb{P}}\left( {{\text{T}},{\text{x}}} \right) \cdot {\text{N}}\left( {\text{t}} \right)\left( {1 - \frac{{{\text{N}}\left( {\text{t}} \right)}}{K}} \right)$$

was used to model the population growth of infected individuals, where *N*(*t*) is the number of MB+ individuals at time *t, F* represents the fecundity, *r* the sex ratio, ${\mathbb{P}}\left( {T,x} \right)$ the probability of infection, survival, and pupation under temperature *T*, and *K* the carrying capacity^74,75^. The carrying capacity was set to 1000 to simulate real-world limitations such as resource and space constraints, establishing a stable population maximum that aligns with natural conditions. Additionally, targeting a population of 1000 MB+ offspring provides a measurable endpoint for assessing the spread *of Microsporidia MB* within mosquito populations. The solution to this equation,

$${\text{N}}\left( {\text{t}} \right) = \frac{K}{{1 + \left( {\frac{{K - {\text{N}}_{0} }}{{{\text{N}}_{0} }}} \right){\text{e}}^{{ - {\text{F}}.{\text{r}}.{\mathbb{P}}\left( {{\text{T}},{\text{x}}} \right).t}} }}$$

enabled us to estimate the rate at which the population of MB+ offspring increases from an initial population of 10 MB+ female *An. arabiensis*, with the goal of reaching a target population of 1000 MB+ individuals.

In our deterministic simulation, parameters such as*: F* (fecundity*), r* (sex ratio), *K* (carrying capacity), and $N_{0}$ (initial population) remained constant. Fecundity was set at three fixed rates (33, 66, or 99 viable eggs per female *An. arabiensis*) based on observed averages, providing a baseline for population growth under stable conditions. The sex ratio male: female was considered to be 1:1. Details of the stochastic simulation are provided in the supplementary material.

To implement this methodology, we used Python for all data processing, simulations, and statistical computations. Python’s libraries, including *numpy* for numerical operations, *scipy* for probability computations and fitting, and *matplotlib* for visualization, were integral to generating plots, calculating probabilities, and fitting model parameters.

## Supplementary Information

Supplementary Information.

## Data Availability

All datasets generated, used and analysed in this study are included in this published article.
