# Supplementary material for: The dissemination potential of Microsporidia MB in Anopheles arabiensis mosquitoes is modulated by temperature
Source: Sci Rep. 2025 Aug 7;15:28839. doi: 10.1038/s41598-025-07414-7 (PMC12328603; doi:10.1038/s41598-025-07414-7)
Supplement: Supplementary file 1 — Supplementary Information. [file 41598_2025_7414_MOESM1_ESM.docx]

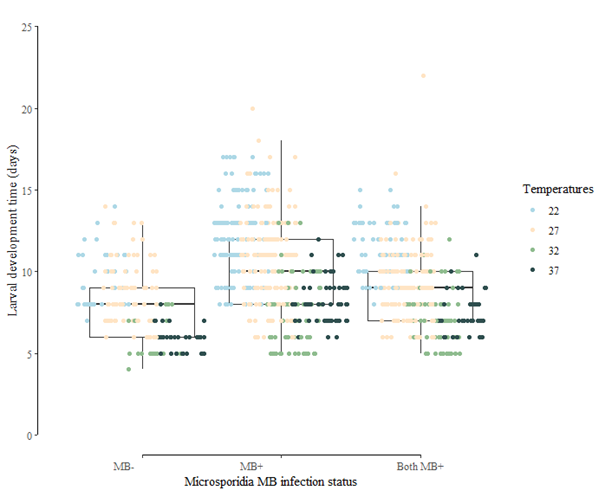


**Supplementary figure 1:** *An. arabiensis* that were MB^+^ due to maternal transmission increased the chance to pupate more than one days faster compared to uninfected larvae coming from MB^+^ female *An. arabiensis* (MB^+^ offspring of MB^+^ female *An. arabiensis* only: 10.2 (9.93-10.52) days; MB^+^ female *An. arabiensis* and offspring: 9.1 (8.85-9.40) days (χ^2^= 50.92, df = 2, p < 0.001), especially at the temperature treatment 27°C (MB^+^ female *An. arabiensis* only: 11.1 (10.66-11.46) days; both MB^+^: 9.4 (9.06-9.83) days; Tukey: p _MB+ G0 female_*_An. arabiensis_* = 0.02; χ^2^= 21.92, df = 6, p = 0.001). At 22°C, 32°C, and 37°C, all larvae coming from MB+ *An. arabiensis* had similar development times (all Tukey test p > 0.05)


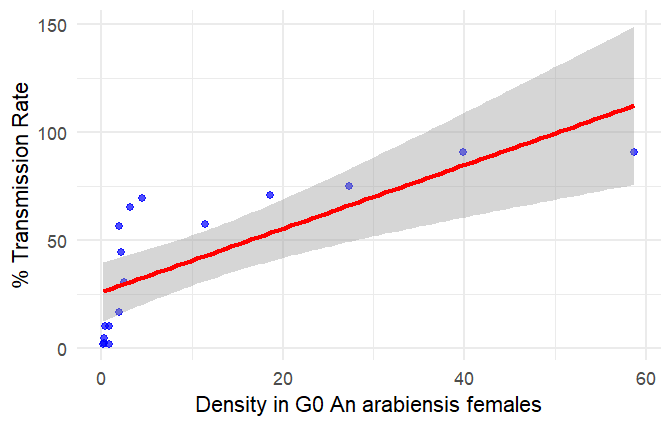


**Supplementary figure 2:** The infection rate in the F1 offspring was positively correlated with the density of *Microsporidia MB* in the G_0_ female *An. arabiensis* (y= 26.09 + 1.47, r^2^ = 0.5612) (χ^2^= 19.18, df = 1, p < 0.0005).


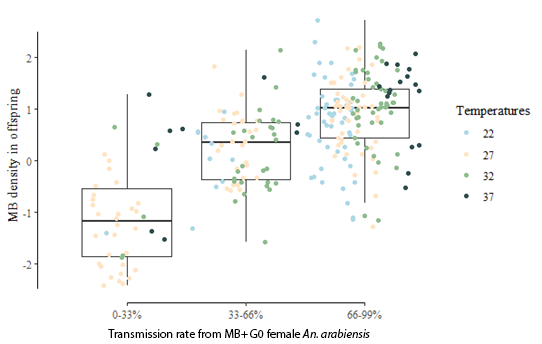


**Supplementary figure 3:** When G_0_ female *An. arabiensis* had a transmission rate higher than 50%, the *Microsporidia MB* density in F1 offspring was higher (between 0 and 33%: 0.08 (0.021-0.144); between 33% and 66%: 0.33 (0.216-0.445); between 66% and 99%: 0.85 (0.734-0.981); (Tukey: p_low-medium_ and p_low-high_ < 0.001, p_medium-high_ = 0.001; χ^2^= 162.66, df = 2, p < 0.001).

- **Stochastic Simulation**

To introduce realistic variation in fecundity, we allowed fecundity rates to fluctuate around the average by approximately ±10%. Each simulation run selected a slightly different fecundity rate based on a normal distribution. This approach means that while the average fecundity rate remains our central value, individual rates vary within a small range to reflect natural differences that might occur in real conditions. To ensure statistical robustness, we conducted 1000 Monte Carlo simulations with this stochastic variation, enhancing the model's predictive power


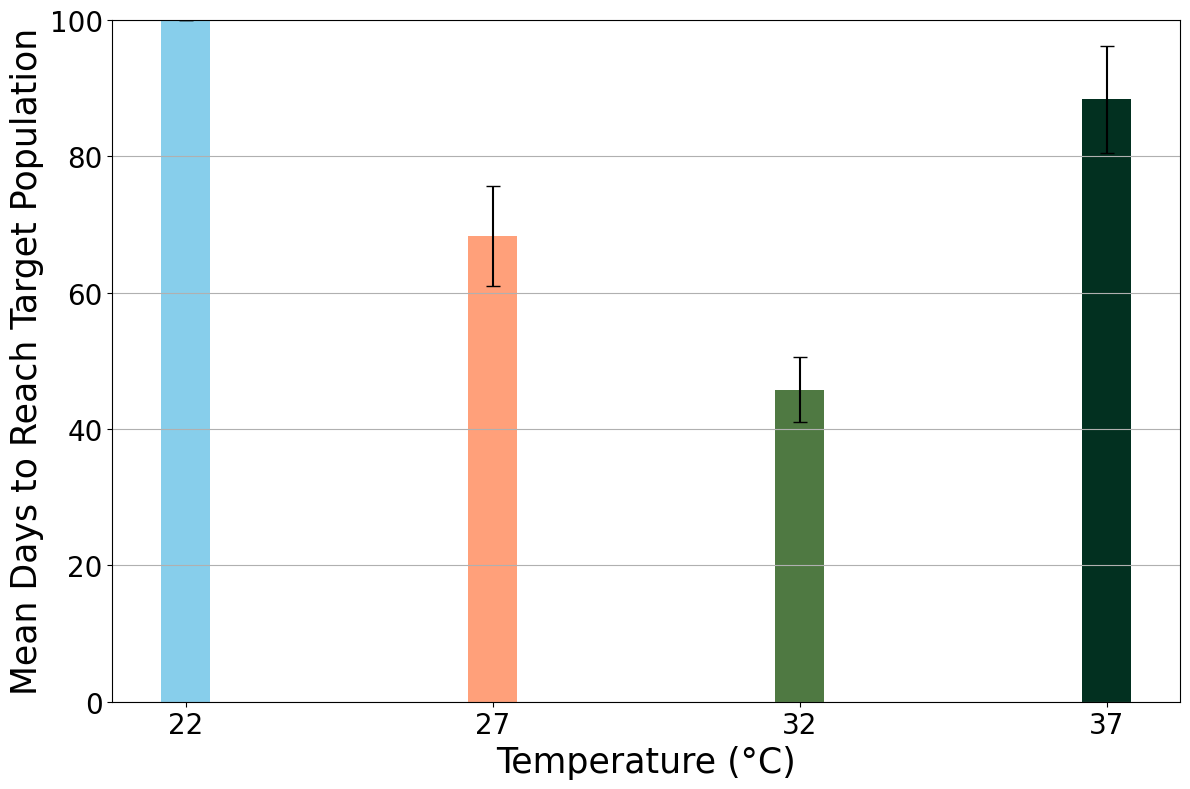


**Supplementary figure 4**: Mean Days to Reach Target Population of 1000 Mothers with Monte Carlo Simulations Across Different Temperatures (fecundity = 99 offspring per female). This plot shows the average number of days required for an infected offspring population to reach a target size of 1000 offspring under varying temperature conditions, based on 1000 Monte Carlo simulations. Starting with an initial population of G_0_ female *An. arabiensis*, each simulation introduces 10% random variability in fecundity to model natural variation, with an average fecundity of 99 offspring per female and a sex ratio of 0.5. For each temperature (22°C, 27°C, 32°C, and 37°C), Gaussian parameters A, mu, and sigma) reflect age-based pupation probability, influencing effective growth rates alongside the fecundity and sex ratio. Each bar represents the mean time to reach the target, with error bars showing standard deviations across simulations, demonstrating how temperature significantly impacts population growth speed and variability.

Link to the code used in modelling <https://colab.research.google.com/drive/1WrqpWw_8QvrL1B0H3HkhEhsbIbMJanr6?usp=sharing>
